# 

**Published:** 1895-12-28

**Authors:** 


### CONTENTS.

**The Andrew Clark Memorial** - - - - - - - - 209

**The Multiplication of Vaccines** - - - - - - 210

**The Treatment of Aneurism.** By A. Pearce Gould, F.R.C.S. - - - - - - - - - - 211

**Medical Progress and Hospital Clinics—**

Three Cases of Diphtheria Treated with Ruffer's Curative Serum - - - - - - - - - - 215

Some Observations on Typhoid Fever - - - - - 216

Progress in Medicine.—Diseases of the Lungs and Pleuræ - 217

Renal Medicine - - - - - - - - - - - 219

New Appliances and Things Medical - - - - - 220

**Modern Sociology.**—In Praise of High Feeding - - - 212

**Annotations.**—A Knavery Extirpation Society—The Chelsea Hospital for Women—Charity and the Press - - - - 213

**Notes and News** - - - - - - - - - - 214

**The Institutional Workshop—**

A Hospital for Lepers in Iceland - - - - - - - 221

Hotel-Dieu du Creusot - - - - - - - - - 221

A New Hospital for Valparaiso - - - - - - - 221

Practical Departments - - - - - - - - 222

**Scraps and Gleanings** - - - - - - - - - 223

**The Book World of Medicine and Science** - - - - 223

### EXTRA SUPPLEMENT.—THE NURSING MIRROR.

**News from the Nursing World.**—Boys at Middlesex Hospital—Entertainments at the Royal Free Hospital—An Important Conference—New Hospital for Women—Newtownards Nursing Society—A Nurse for Chard Workhouse—District Nursing for Huntingdon and Godmanchester—Glasgow Nurses' Co-operation—The Arbroath Association—New Rooms for Parisian Nurses—Christmas Greeting—Wallsend Nursing Association—Private Nurses in San Francisco—A Plea from Ireland—The Average Value of Certificates—Intermittent Training—District Nurses in Lancashire—Short Items - - - - - - ciii
**Lectures on Nursing.** II.—Health, Method, Sympathy - - cv
**Food, with Special Relation to the Sick.** I.—Administration of Nourishment - - - - - - - - - cvi
**"Truth" Doll and Toy Show** - - - - - - - cvi
**Our Needlework Competition** - - - - - - - cvii
**Novelties for Nurses** - - - - - - - - - cvii
**Everybody's Opinion** - - - - - - - - - cviii
**First Aid at Aldershot** - - - - - - - - cviii
**St. Catherine's Home, Bradford** - - - - - - cviii
**A Tribute to Medical Women** - - - - - - - cviii
**Where to Go** - - - - - - - - - - - cviii
**Notes and Queries** - - - - - - - - - - cviii

